# Connecting Dynamics and Thermodynamics in Polymer–Resin Cured Systems

**DOI:** 10.3390/polym16243508

**Published:** 2024-12-17

**Authors:** Luis A. Miccio, Clemens Sill, Carsten Wehlack, Gustavo A. Schwartz

**Affiliations:** 1 Centro de Física de Materiales (CSIC-UPV/EHU)-Materials Physics Center (MPC), P. M. de Lardizábal 5, 20018 San Sebastián, Spain; 2 Donostia International Physics Center, P. M. de Lardizábal 4, 20018 San Sebastián, Spain; 3 Institute of Materials Science and Technology (INTEMA), National Research Council (CONICET), Colón 10850, Mar del Plata 7600, Buenos Aires, Argentina; 4 Goodyear Innovation Center, Goodyear S.A., L-7750 Colmar-Berg, Luxembourg

**Keywords:** elastomers and rubbers, calorimetry, dielectric spectroscopy, modelling dynamics, polymer–resin interaction

## Abstract

This work connects the calorimetric responses of different rubber–resin blends with varying resin contents with their alpha relaxation dynamics. We used differential scanning calorimetry and broadband dielectric spectroscopy to characterize the calorimetric and dielectric responses of styrene–butadiene, polybutadiene, and polyisoprene with different resin contents. To model the results, we used the Gordon–Taylor equation combined with an extension of the Adam–Gibbs approach. Thus, we propose a simple and effective model that allows us to estimate the blend dynamics from the temperature dependence of the relaxation times of the pure components and the calorimetric measurement of the glass transition temperature of only one blend composition. By estimating an effective interaction parameter from calorimetry, we achieved accurate alpha relaxation dynamics predictions for different resin concentrations. Our highly predictive approach provides a realistic description of the expected dynamics. This study offers valuable insights into the dynamic properties of polymer compounds, paving the way for the fast and effective development of advanced and more sustainable materials.

## 1. Introduction

Rubber-based compounds, particularly in the tire industry, have shown significant advancements over the years. One key area of development has been the blending of synthetic rubber polymers, such as styrene–butadiene rubber (SBR), polyisoprene (PI), and polybutadiene (PBD) [1,2,3,4,5]. These materials have improved tires’ performance and their resistance to wear and tear. In addition to synthetic rubber polymers, various fillers and oligomers have been progressively incorporated into tire compounds to enhance their properties. For example, silica and other fillers have been used to improve tire traction and wet grip [6,7,8,9,10,11,12,13,14]. Recent scientific achievements in the rubber industry have focused on developing new formulations that can enhance the performance of tires while reducing their environmental impact [15,16,17,18].

Among other strategies, adding resins to rubber compounds has been recently studied as a promising way of enhancing performance and reducing production costs [19]. Resins, also known as tackifiers, can enhance the adhesion between rubber and other materials, leading to stronger and more durable materials. They can also improve cohesion properties, making rubber compounds more flowable and easier to process, resulting in more efficient manufacturing and lower costs. Moreover, resins can reduce the amount of synthetic rubber polymers required in the compound. However, optimal selection of the type and quantity of resin is necessary for achieving the best balance between performance and cost, and further research in this area is required. In particular, polymer–resin interactions can lead to homogeneous or heterogeneous materials [20,21], and they define how properties change with resin concentration. For these reasons, it is necessary to understand how the resin interacts with the rest of the components to reach the desired compound properties [22,23,24].

In previous studies, several extensions of the Adam−Gibbs (AG) approach were proposed to account for the segmental dynamics of polymers, polymer blends, and polymer/plasticizer mixtures under different conditions [25,26,27,28,29]. More recently, we have proposed a further extension of the AG approach to include the effects of cross-links in vulcanized rubber blends [29]. In this study, we focus on estimating the polymer–resin interaction parameter [30] and its impact on the dynamics of rubber compounds modified with resins. Specifically, we compare the effect of a hydrogenated DCPD/C9 resin on three different polymers, namely, polybutadiene rubber, styrene−butadiene rubber, and polyisoprene, using a simple method based on the extended Adam–Gibbs [25,26,29,31] approach and the Gordon–Taylor equation. Our findings will contribute to connecting the thermodynamics of mixtures with the observed dynamics and to a more in-depth understanding of the role of resins in rubber compounds, as well as provide accurate predictions with only a calorimetric measurement on one blend composition.

## 2. Materials and Experimental Methods

### 2.1. Materials

We investigated the dynamics of miscible blends of polybutadiene rubber (PBD), styrene−butadiene rubber (SBR), and polyisoprene (PI) with various concentrations of a hydrogenated DCPD/C9 resin with an aromatic level of 10%. A summary of the composition of the different blends is shown in Table 1.

### 2.2. Sample Preparation

Rubber compounds were prepared by mixing polymer, resin, and additives in a lab-scale internal mixer. Sulfur and cure additives were added in the second stage. Cured square sheets measuring 100 × 100 × 0.7 mm^3^ were obtained by curing the compounds in a press at 170 °C for 10 min. Samples for dielectric and calorimetric measurements were punched out from the cured sheets using a circular die of 40 mm and 5 mm in diameter for BDS and DSC experiments, respectively.

### 2.3. Modulated Differential Scanning Calorimetry (MDSC)

**Figure 1:**
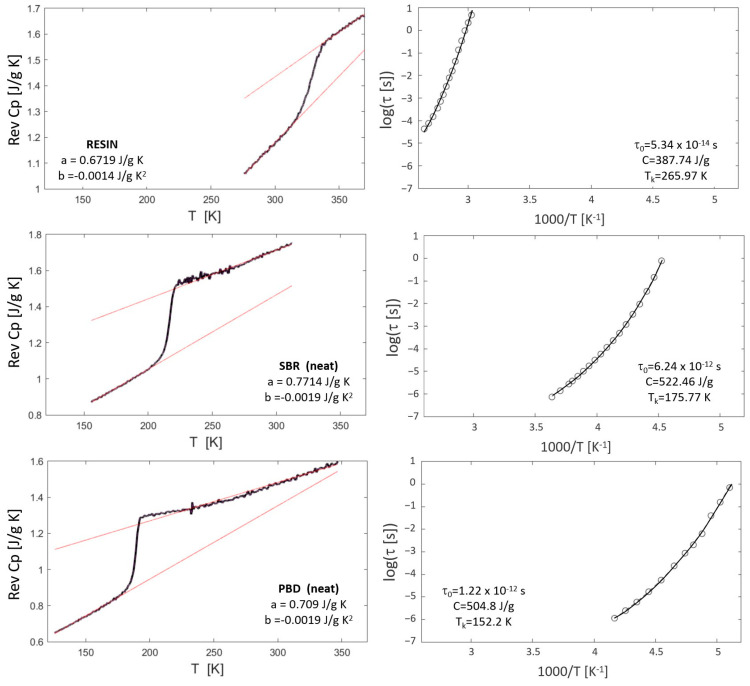

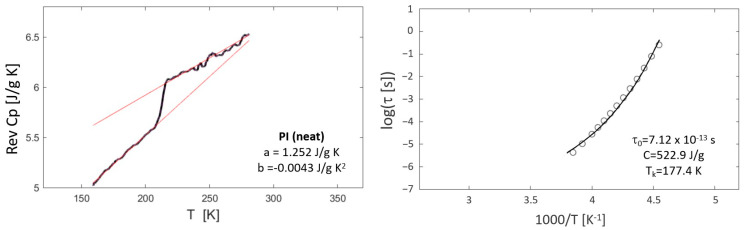
Left column: DSC thermograms (reversible C_p_ vs. temperature) of pure resin and neat SBR, PBD, and PI. Red lines represent the linear fittings for $∆C_{p}(T)$ determination. Right column: experimental data (circles) of the temperature dependence of the relaxation time and corresponding Adam–Gibbs fittings (lines). The obtained parameters are shown in the plots.

Calorimetric measurements were performed using a Q2000 TA Instruments DSC (New Castle, USA) in modulated mode, with a 60 s period and 0.5 K amplitude. The underlying heating rate ranged between 0.5 and 3 K/min. Modulated heating and cooling cycles were conducted under nitrogen flow in the temperature range from 100 to 400 K. Samples weighing between 5 and 10 mg were sealed in hermetic aluminum pans. The MDSC technique separates the total heat flow into reversible and non-reversible components based on the system’s response to a changing heating rate. This method allows for accurate heat capacity (Cp) measurements by isolating reversible Cp signals associated with changes in heat capacity and melting. The glass transition temperature (*T_g_*) was determined at the inflection point of the reversing curve. The heat capacity change at *T_g_* was estimated from the extrapolation of the Cp behavior well above and below *T_g_* (see red lines in DSC thermograms in Figure 1).

### 2.4. Broadband Dielectric Spectroscopy (BDS)

Dielectric measurements were performed using a Novocontrol Alpha high-resolution broadband dielectric spectrometer in the 10^−1^–10^6^ Hz frequency range. Disk-shaped samples with a thickness of approximately 0.7 mm and gold-plated electrodes with diameters of 40 mm were used for the measurements. For the parallel-plate configuration, the sample capacitance is related to the dielectric permittivity through the equation *C* = *εε*_0_*A*/*d*, where ε is the relative dielectric permittivity of the sample, ε_0_ is the vacuum permittivity, *A* is the area of the sample, and *d* is the sample thickness. We measured the complex dielectric permittivity, ε*, which is defined as ε*(*ω*) = C*(*ω*)/*C*_0_ = *ε′*(*ω*) − *i ε″*(*ω*), where *C*_0_ is the capacitance of the empty capacitor and *ω* = 2*πf* [32]. Isothermal frequency scans were performed at intervals of 3 to 5 degrees over the temperature range of 120–400 K. The sample temperature was controlled using nitrogen gas flow with temperature stability better than ±0.1 K.

The obtained spectra were analyzed using several Cole–Cole (CC) functions to fit the different relaxation processes [32]. The conductivity effects, observed as a low-frequency contribution to the dielectric loss, were accounted for by adding a conductivity term. Therefore, the complex dielectric permittivity was modeled as:

(1) $$\varepsilon^{*}\left(\omega \right)-\varepsilon_{\infty }=\frac{∆\varepsilon }{1+{\left(i\;\omega \;\tau_{CC}\right)}^{\alpha }}-i\frac{\sigma }{\varepsilon_{0}\omega }$$

where $\varepsilon_{\infty }$ is the high-frequency permittivity, Δε is the dielectric strength, $\tau_{CC}$ is the relaxation time, ω is the angular frequency, α is a shape parameter that accounts for the symmetric broadening of the relaxation peak, and σ is the dc conductivity. The main (*α*) and secondary (*β*) relaxation processes were fitted separately to capture the dynamics of the segmental motions and localized movements, respectively. Finally, relaxation maps were constructed by plotting the logarithm of the alpha relaxation times ($\log\tau_{CC}$) as a function of inverse temperature (1000/T [K^−1^]).

## 3. Theoretical Considerations

The Adam–Gibbs (AG) theory [33] relates increases in structural relaxation time (*τ*) to the reduction of configurational entropy (*S_c_*) according to the following equation:

(2) $$\tau (T)=\tau_{0\;}\;e^{\left(\frac{C_{0}}{TS_{c}}\right)}$$

where *τ*_0_ is the relaxation time at very high temperatures and *C*_0_ is a constant that depends on the material. The configurational entropy is not experimentally accessible and is usually estimated from the excess entropy (*S_ex_* = *S_melt_* − *S_crystal_*). Thus, *S_c_* can be written as follows [25,26]:

(3) $$S_{c}\left(T\right)\approx \;gS_{ex}\left(T\right)=g\int_{T_{k}}^{T}\frac{\Delta Cp(T’)}{T}dT’$$

where ∆*Cp* is the excess heat capacity at temperature *T*, which can be approximated by $∆C_{p}=a+bT$ (see Figure 1); *T_k_* is the Kauzmann temperature; and *g* is a proportionality constant between *S_ex_* and *S_c_* that depends on the material. Therefore, it is possible to obtain the corresponding parameters by combining DSC and BDS measurements. After integrating *S_c_*(*T*), *a* and *b* are obtained from fitting the experimental DSC curves (see Figure 1) and replaced in the *τ*(*T*) (Equation (2)), resulting in the following expression:

(4) $$\tau (T)=\tau_{0\;}\;exp\left\{\frac{C}{T\left[a\;ln\left(\frac{T}{T_{k}}\right)+b\;(T-T_{k})\right]}\right\}$$

where $C=C_{0}/g$ is a constant that depends on the material. It is worth noting that the reported a and b parameters arise from the difference between the two individual linear fittings in Figure 1. Therefore, although $C_{p}$ has a positive slope for all the studied samples, $∆C_{p}$ presents negative values since the $C_{p}$ of the melt increases slower than that of the glass. For more details about the AG extended approach, the reader can look at previous works [26,29,34].

In the case of blends of two components, mixing rules must be applied to calculate the excess entropy of the mixture. In our approach, we assume that the cured polymer (neat) and the resin are the two components of the blends and that their effective concentrations correspond to their macroscopic ones [29]. Following previous works [26,29,34], we only include the interaction term for calculating the specific excess entropy in the blend, whereas for the parameters $C^{blend}$ and $\tau_{0}^{blend}$ the interactions can be neglected. In addition, we assume, as a first-order approximation, that the interaction parameter $\left(\chi_{eff}^{AG}\right)$ does not depend on temperature nor on concentration. The resulting expressions are as follows:

(5) $$C^{blend}=w_{1}C_{1}+w_{2}C_{2}$$

(6) $$\tau_{0}^{blend}=w_{1}\tau_{0_{1}}+w_{2}\tau_{0_{2}}$$

(7) $$S^{blend}=w_{1}S_{1}+w_{2}S_{2}+w_{1}w_{2}\chi_{eff}^{AG}$$

(8) $$\gamma =\frac{w_{1}C_{1}+w_{2}C_{2}}{T\;\left\{w_{1}\left[a_{1}\;ln\left(\frac{T}{T_{k_{1}}}\right)+b_{1}\;(T-T_{k_{1}})\right]+w_{2}\left[a_{2}\;ln\left(\frac{T}{T_{k_{2}}}\right)+b_{2}\;(T-T_{k_{2}})\right]+w_{1}w_{2}\chi_{eff}^{AG}\right\}}$$

(9) $${log(\tau }^{blend})=\log\left(\tau_{0}^{blend}\;\right)+\mathrm{\gamma log}\left(e\right)$$

where $w_{1}$ and $w_{2}$ stand for the mass fractions of the polymer and the resin, respectively, and $\chi_{eff}^{AG}$ represents the effective interaction between the polymer and resin.

In previous works [29], the parameter $\chi_{eff}^{AG}$ was calculated by fitting the temperature dependence of the relaxation times. However, one possible way of estimating this parameter is through the Gordon–Taylor equation [35,36] (see Equation (10)). The Gordon–Taylor approach (GT) is a widely used method for estimating the glass transition temperature of blends and mixtures, in which the key concept is that the *T_g_* of the blend can be estimated based on a relationship between the *T_g_* of the pure components, their weight fractions, and a fitting parameter known as $k_{GT}$ [35,36,37,38,39,40]. More importantly, this last parameter can be explicitly related to the interaction parameter $\left(\chi_{eff}^{GT}\right)$ and the heat capacities [35] (see Equation (11)).

(10) $$T_{g}^{blend}=\frac{T_{g1}w_{1}+T_{g2}w_{2}k_{GT}}{w_{1}+w_{2}k_{GT}}$$

(11) $$k_{GT}\approx \;\frac{\Delta {Cp}_{2}}{\Delta {Cp}_{1}}-\frac{\chi_{eff}^{GT}}{\Delta {Cp}_{1}}$$

Finally, we assume that both effective interaction parameter values are approximately equal; this means that $\chi_{eff}^{GT}\approx \chi_{eff}^{AG}$, hereafter called $\chi_{eff}$. In this way, the GT expression allows for estimating the effective interaction parameter used in the mixing rule of the extended AG approach directly from calorimetric measurements. It is worth noticing that our approach assumes a constant interaction parameter value. Therefore, its accuracy may be reduced under extreme conditions where this parameter exhibits significant temperature and/or concentration variations. If this is the case, an additional parameter can be added to consider such variations. However, the first-order approximation we propose here works well in most cases. As a result, the alpha relaxation dynamics of the mixtures at any temperature and concentration can be estimated from dielectric and calorimetric data of the pure components, plus a sole calorimetric experiment to know the *T_g_* of the blend at a single composition.

## 4. Results and Discussion

To apply the proposed approach to describe the segmental dynamics of polymer–resin blends, the dynamics and thermodynamics of the neat components need to be fully characterized using Equation (4). Once this step is performed, the second step is to estimate the effective interaction parameter for each system from the calorimetric measurements of the blends. Finally, the third step consists of using the calculated parameters to predict the dynamics of the blends at any temperature and composition using the extended AG approach (Equation (9)). We finally validated this method by comparing the predictions with the experimental dynamics obtained from the BDS measurements.

### 4.1. Characterization of the Neat Components

We started by measuring the calorimetric response of the neat components, as described in Section 2.3. The results are shown in Figure 1 (left column), where the red lines show the linear fitting of the temperature dependence of the reversible heat capacity below and above the glass transition temperature. From these fittings, the corresponding $∆C_{p}$ values as a function of the temperature were calculated. The obtained values of the parameters *a* and *b* are shown in Figure 1 (left column).

On the other hand, the dielectric response of the neat components was measured as described in Section 2.4. The relaxation time, obtained from fitting the imaginary part of the complex dielectric permittivity, was plotted as a function of inverse temperature. Figure 1 (right column) shows the corresponding relaxation maps where circles represent the experimental data. From these plots, Equation (4) can be used to fit the temperature dependence of the segmental relaxation times. Solid lines in Figure 1 (right column) show the excellent agreement between the experimental data and the AG model for neat resin and unfilled SBR, PBD, and PI. The corresponding fitting parameters for all neat compounds are shown in Figure 1.

### 4.2. DSC Measurements of Polymer–Resin Systems

Once the full characterization of the neat components has been performed, we can explore the ability of the proposed model to describe the dynamics of polymer–resin blends. Equation (8) provides the temperature dependence of the segmental relaxation time for each blend, whereas the corresponding parameters are those shown in Figure 1. Since the dynamics of the neat components and the concentration in the blends are known, only the interaction parameter ($\chi_{eff}^{AG}$) is necessary to fit the dynamics of the different blends at any temperature and concentration.

**Figure 2:**
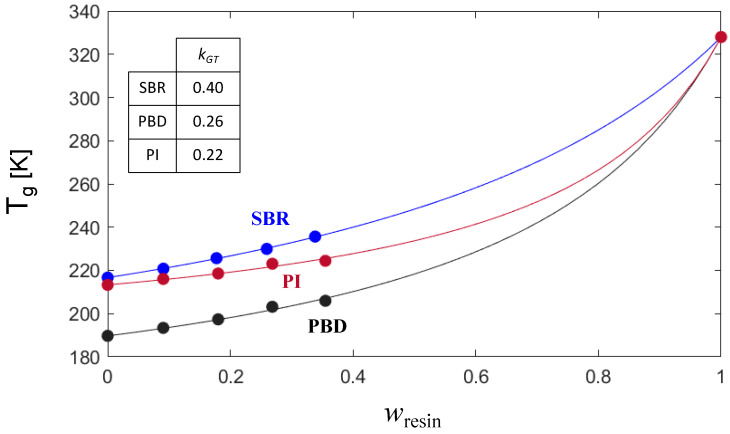
Gordon–Taylor fitting of the resin concentration dependence of the *T_g_* for the SBR-, PBD-, and PI-based systems. $k_{GT}$ stands for the GT parameter as obtained from the fitting.

Figure 2 shows the polymer–resin blends’ glass transition temperature dependence on the resin content. The neat polymers were plotted as component 1 ($w_{resin}=0$) and the resin was plotted as component 2. For the three studied systems (SBR, PBD, and PI) a non-linear dependence of their calorimetric glass transition with varying resin concentration is observed. The lines in Figure 2 show the fitting of the experimental values using the GT approach (Equation (9)). Once the $k_{GT}$ parameter is obtained from the fittings, it is possible to use Equation (10) to calculate the interaction parameter $\chi_{eff}^{GT}$. The corresponding obtained values are shown in Figure 2.

**Figure 3:**
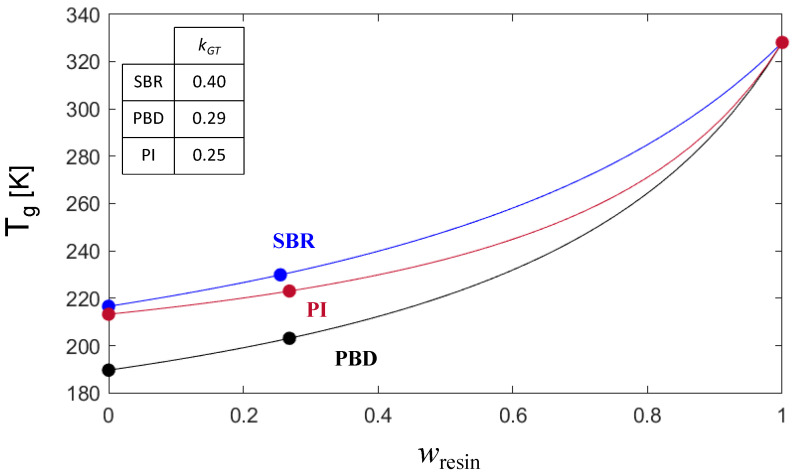
Gordon–Taylor fitting of *T_g_* for the SBR-, PBD-, and PI-resin blends using only a single blend composition (25% resin).

It is worth noting that we can take full advantage of the proposed method, in terms of time and resources, to estimate $\chi_{eff}^{GT}$ from the GT fitting by using only one blend composition. We have found minor differences for each selected combination of samples (i.e., when using pure components and one of the blend experimental compositions). We propose the use of intermediate concentrations for the calculation of the $k_{GT}$; for instance, 25.5 or 34% resin in our case. Figure 3 presents the obtained $k_{GT}$ estimations for each system by employing this procedure using the *T_g_* of the pure components and the 25.5% resin sample. As observed, the differences with the complete fitting are around 10%.

### 4.3. Compound Dynamics from BDS

**Figure 4:**
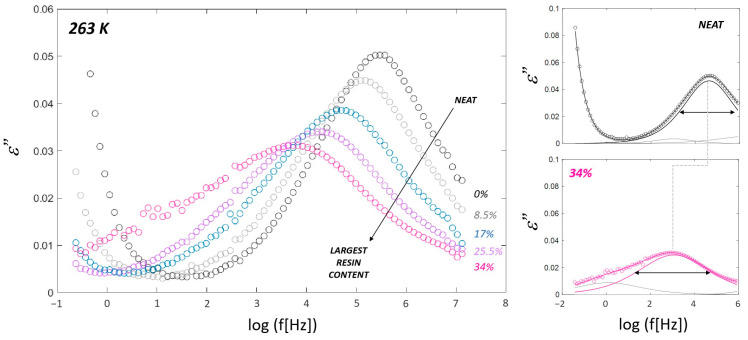
(**Left**) Imaginary part of the complex dielectric permittivity as a function of frequency at 263 K for the SBR-based system with different resin concentrations. The arrow indicates increasing resin content. (**Right**) Experimental dielectric response (circles) and the corresponding fittings (lines) for SBR–resin samples with 0 and 34% resin. The dashed line intends to show the frequency shift of each alpha relaxation (alpha relaxation peaks are indicated in the sample’s color, whereas other contributions are presented in grey), and the width at half maximum is illustrated by the black horizontal arrows.

The alpha relaxation process of the polymer–resin blends was studied using broadband dielectric spectroscopy (BDS). Figure 4 shows the imaginary part of the complex dielectric permittivity $\left(\varepsilon ”\right)$ as a function of log (f[Hz]). As shown in the Figure, the effect of the resin can be easily noticed in the spectra, as the frequency of the main process decreases upon increasing resin content. In addition, the broadness of the peak increases with increasing resin content. This behavior, commonly observed for miscible blends, is related to the increase in the mixture’s heterogeneity [4,41].

Figure 4 (right column) shows the individual contributions obtained from the fitting process for both the neat SBR and the 34% SBR–resin samples at 263 K. The dielectric spectra are accurately described in both cases using three Cole–Cole functions. These individual contributions correspond to, from high to low frequencies, the beta and alpha relaxations of the main polymer and a slow relaxation process (which is attributed to the accelerator used for curing [2]). For the neat polymer (see black line), the alpha relaxation peak is observed at higher frequencies, reflecting faster molecular dynamics, as DSC measurements corroborate. In the 34% resin sample (see pink line), the alpha relaxation peak shifts to lower frequencies, indicating slower dynamics caused by the resin’s restrictive effect on polymer mobility. The broadening of the peaks (as represented by horizontal arrows depicting the full width at half maximum) highlights the increased distribution of relaxation times, which is linked to the heterogeneous environments introduced by the resin.

**Figure 5:**
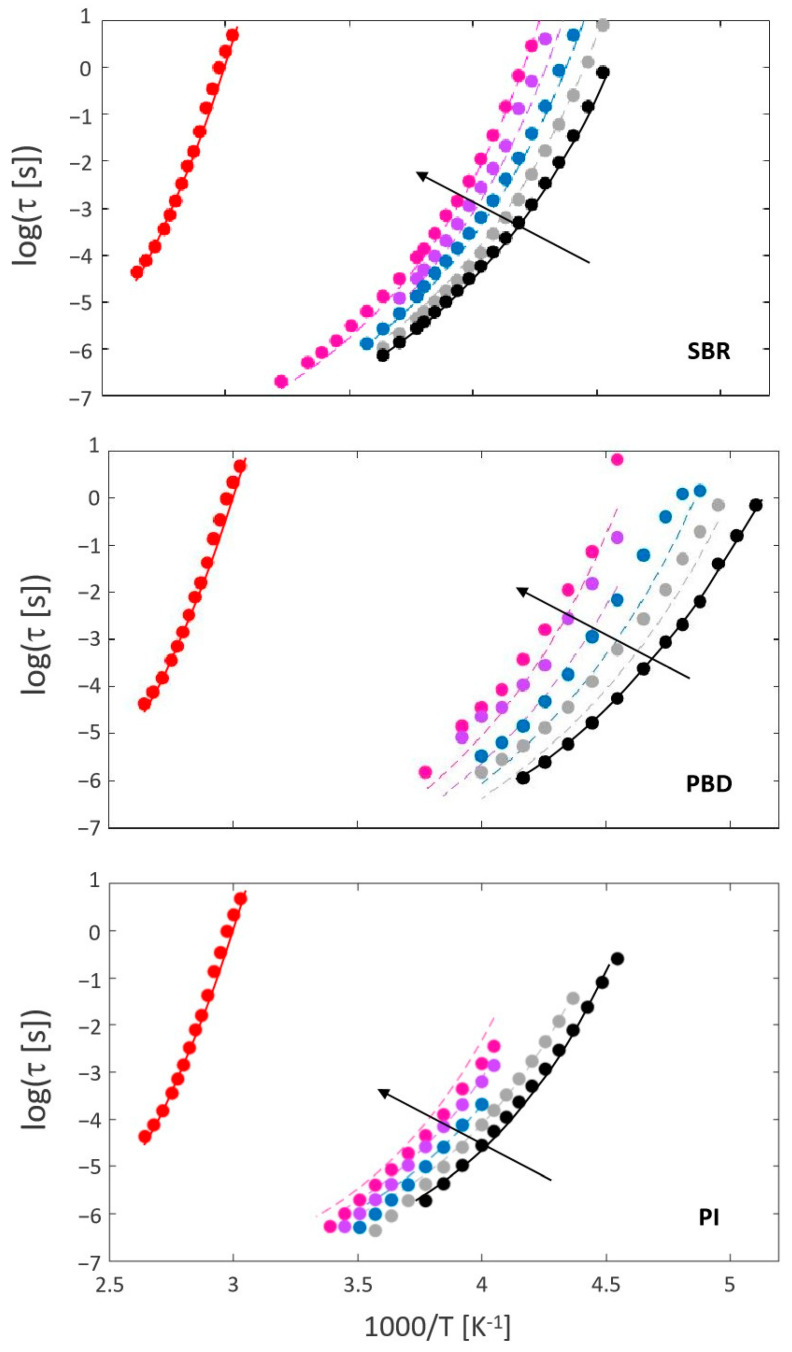
Log τ as a function of 1000/T for SBR-, PBD-, and PI-resin based systems’ alpha relaxation. The arrow indicates increasing resin content for the compounds (the neat and the pure resin are also included in the map).

Figure 5 shows the relaxation maps for resin (red dots), neat polymers (black dots), and polymer–resin blends at different compositions. The Figure shows how the structural relaxation time becomes slower with increasing resin content, which is consistent with the results of the thermal characterization (see Figure 2).

### 4.4. Blend Dynamics Estimation and Correlation with Experimental Results

The AG approach has been effectively implemented to describe the temperature dependence of the structural relaxation time of polymers, polymer blends, and polymer–plasticizer systems under several conditions [27,28,29,34]. As mentioned in previous sections, the extended AG approach describes the dynamics of blends by combining the model parameters of the pure components (see Figure 1) with a non-ideal mixing rule (see Equation (8)) for the excess entropy. Polymer–resin interactions account for the non-ideal behavior and are incorporated using an effective interaction parameter. In this approximation, we use a constant *χ_eff_* to predict the system’s dynamics, regardless of temperature and concentration. The obtained results are shown in Figure 5. The agreement between the experimental data (circles) and the AG approach (lines) is quite accurate, particularly considering that only one resin concentration was used to estimate *χ_eff_*.

**Figure 6:**
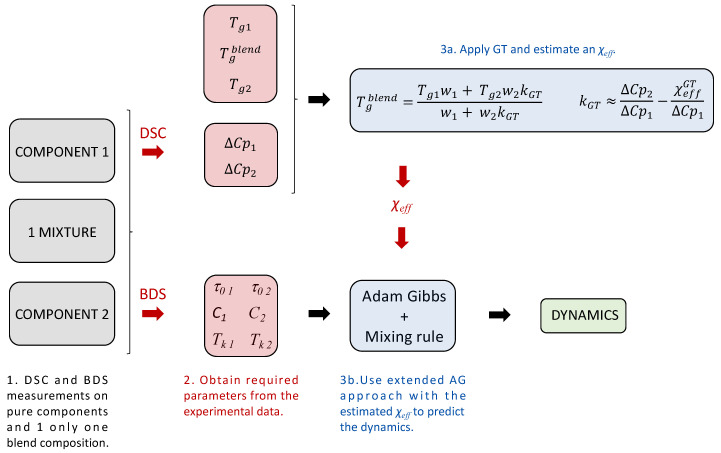
Dynamics prediction flowchart.

Figure 6 presents a schematic picture of the calculations and the experimental data needed to obtain the prediction of the temperature dependence of the structural dynamics for a given polymer–resin blend. In the first step, samples of the neat components (resin and cured polymers) are measured by DSC and BDS. In addition, a single blend composition is also measured by DSC. In the second step, the glass transition temperatures $\left(T_{g_{1}},T_{g_{2}},T_{g}^{blend}\right)$, the excess heat capacities $\left(∆C_{p}=a+bT\right)$, and the AG parameters $\left(\tau_{0},C,T_{k}\right)$ are obtained from the fitting of the experimental data of the neat components. In the last step, the resin composition dependence of the glass transition temperature is fitted with the GT approach, the *χ_eff_* is estimated, and the structural dynamics are predicted using the extended AG approach. The results are shown in Figure 5 (dashed lines), together with the corresponding experimental data.

Besides the full characterization of the two neat components, it is noteworthy that only a single DSC measurement is necessary to predict the blend dynamics. This provides a shorter and cheaper way of estimating the compound’s behavior with varying resin content with a minimum experimental effort. Thus, the proposed approach contributes to accelerating the development of new materials, saving time and resources, and reducing production costs.

## 5. Conclusions

In this study, we propose a novel approach that combines the Gordon–Taylor equation with an extension of the Adam–Gibbs model to predict the dynamics of several polymer–resin blends. In this way, we were able to estimate the interaction parameter between the two components in the blend from a single calorimetric measurement, with which we obtained a realistic description of the blends’ dynamics for different resin concentrations. We also provided a good description of the experimental thermal and dielectric responses of the blends with a wide range of resin concentrations and temperatures, which indicates the robustness and versatility of our model. This approach provides meaningful insights into the polymer blends’ dynamics and exhibits high predictive capabilities.

## Figures and Tables

**Table 1:** Composition summary of the compounds used in this study. The Polymer column refers to the base material (SBR, PBD, or PI). The columns labeled pol, add, and resin show the phr (per hundred rubber) content of polymer, additives, and resin in the different compounds. The vol% column refers to the resin volume concentration of each sample.

Polymer	Pol	Add	Resin	vol%
SBR, PBD or PI	100	10.4	0	0
	100	10.4	11	8.5
	100	10.4	24.25	17
	100	10.4	40.5	25.5
	100	10.4	60.75	34
	0	0	100	100

## Data Availability

The data that support the findings of this study are available within the article.
